# Quality and safety of medication use in primary care: consensus validation of a new set of explicit medication assessment criteria and prioritisation of topics for improvement

**DOI:** 10.1186/1472-6904-12-5

**Published:** 2012-02-08

**Authors:** Tobias Dreischulte, Aileen M Grant, Colin McCowan, John J McAnaw, Bruce Guthrie

**Affiliations:** 1 Tayside Medicines Unit, NHS Tayside, Mackenzie Building, Kirsty Semple Way, Dundee, Scotland, DD2 4BF, UK; 2 Population Health Sciences, University of Dundee, Mackenzie Building, Kirsty Semple Way, Dundee, Scotland, DD2 4BF, UK; 3 NHS 24 East Contact Centre, 2 Ferrymuir, South Queensferry, Scotland, EH3 0 9QZ, UK

**Keywords:** Medication error, quality indicator, primary health care, adverse drug events, preventable drug related morbidity

## Abstract

**Background:**

Addressing the problem of preventable drug related morbidity (PDRM) in primary care is a challenge for health care systems internationally. The increasing implementation of clinical information systems in the UK and internationally provide new opportunities to systematically identify patients at risk of PDRM for targeted medication review. The objectives of this study were (1) to develop a set of explicit medication assessment criteria to identify patients with sub-optimally effective or high-risk medication use from electronic medical records and (2) to identify medication use topics that are perceived by UK primary care clinicians to be priorities for quality and safety improvement initiatives.

**Methods:**

For objective (1), a 2-round consensus process based on the RAND/UCLA Appropriateness Method (RAM) was conducted, in which candidate criteria were identified from the literature and scored by a panel of 10 experts for 'appropriateness' and 'necessity'. A set of final criteria was generated from candidates accepted at each level. For objective (2), thematically related final criteria were clustered into 'topics', from which a panel of 26 UK primary care clinicians identified priorities for quality improvement in a 2-round Delphi exercise.

**Results:**

(1) The RAM process yielded a final set of 176 medication assessment criteria organised under the domains 'quality' and 'safety', each classified as targeting 'appropriate/necessary to do' (quality) or 'inappropriate/necessary to avoid' (safety) medication use. Fifty-two final 'quality' assessment criteria target patients with unmet indications, sub-optimal selection or intensity of beneficial drug treatments. A total of 124 'safety' assessment criteria target patients with unmet needs for risk-mitigating agents, high-risk drug selection, excessive dose or duration, inconsistent monitoring or dosing instructions. (2) The UK Delphi panel identified 11 (23%) of 47 scored topics as 'high priority' for quality improvement initiatives in primary care.

**Conclusions:**

The developed criteria set complements existing medication assessment instruments in that it is not limited to the elderly, can be implemented in electronic data sets and focuses on drug groups and conditions implicated in common and/or severe PDRM in primary care. Identified priorities for quality and safety improvement can guide the selection of targets for initiatives to address the PDRM problem in primary care.

## Background

Systematic reviews have demonstrated deficits in the quality and safety of medication use in primary care to an extent sufficient to constitute a public health threat. Three to four percent of all unplanned hospital admissions are due to preventable drug related morbidity (PDRM), with the majority attributed to high-risk prescribing and inconsistent monitoring [1-4]. Antiplatelets, diuretics, non-steroidal anti-inflammatory drugs (NSAIDs) and anticoagulants account for almost half of preventable drug-related admissions to hospital, with opioid analgesics, beta-blockers, drugs affecting the renin angiotensin system and anti-diabetic agents also frequently implicated [1]. In addition, safety alerts have been issued for drugs less commonly implicated in PDRM but associated with preventable deaths, such as prescribing and monitoring of methotrexate [5] and use of antipsychotics in older people with dementia [6]. These figures are likely to underestimate PDRM caused in primary care, since the negative consequences of under-use of effective guideline recommended drugs have not consistently been considered by the hospitalisation studies included in systematic reviews [1-4].

The 'Data-driven Quality Improvement in Primary care (DQIP)' research programme is designing and testing a complex intervention to improve the quality and safety of medication use in UK primary care. It is based on encouraging and facilitating primary care medical practices to systematically and continuously identify, correct or otherwise manage drug therapy risks that are potential pre-cursors to PDRM [7]. The DQIP approach requires explicit medication assessment criteria which can (1) be operationalised in existing UK electronic data sources in order to (2) identify patients at risk of common or severe PDRM in primary care.

A number of explicit medication assessment tools have been developed in recent years. The Beers criteria set [8] lists potentially inappropriate drugs in the elderly and can be relatively easily implemented in electronic data sets. However, a large proportion of listed items are not licensed or rarely used in the UK and many of the drug groups frequently associated with preventable harm are not considered. More recently published tools that also focus on the elderly, such as 'Assessing care of vulnerable elders' (ACOVE) [9], 'Screening Tool of Older Person's Prescriptions (STOPP)' and 'Screening Tool to Alert doctors to Right Treatment' (START) [10] have a broader scope, but many of the included criteria require manual record review and/or clinical judgement, which are barriers to routine or large scale applications. Other instruments that have been implemented in electronic records and target the primary care population at large [11-13] cover a limited spectrum of medication use issues, especially with respect to medication safety.

The study had two aims. First, we aimed to develop and classify by clinical importance a set of up-to-date medication assessment criteria that can be implemented in routine primary care clinical datasets to identify instances of (a) sub-optimally effective medication use for conditions commonly encountered in primary care and (b) high-risk use of drugs that have been shown to either commonly cause harm and/or cause severe harm in primary care. Second, we aimed to elicit the extent to which thematically-related medication assessment criteria, subsequently referred to as *topics*, are perceived to be priorities for quality improvement by professionals working in UK primary care.

## Methods

### Study design

The study was conducted in three stages. First, an extensive list of candidate medication assessment criteria was generated based on a structured literature review. Second, an expert panel participating in a modified RAND/UCLA (University College of Los Angeles) Appropriateness Method (RAM) study scored these items by clinical importance based on a summary of research evidence and their clinical judgement. Candidate criteria with high importance scores were translated into a final criteria set by removing redundancies (see below). Final criteria were characterised by the type of medication use targeted, informed by available taxonomies [13-15]. Third, thematically related final criteria were clustered into medication improvement topics and those derived from candidates with high importance scores were presented to a larger Delphi panel of clinicians working in UK primary care for prioritisation. The study was approved by the Tayside Committee on Medical Research Ethics A (reference no. 09/S1401/54).

### Literature review

Prescribing is a ubiquitous feature of medical care which makes a systematic evaluation of the literature on prescribing quality or safety unfeasible in a single research project. We therefore focussed on medication use for conditions commonly encountered in primary care and drugs with clear evidence of significant benefit or harm. The literature review drew initially on UK national clinical guidelines, prescribing advice, and safety alerts, supplemented by European or other clinical guidelines and targeted primary literature review in selected areas as detailed below.

Candidate medication assessment criteria either described potentially beneficial medication use ('quality') or the use of potentially harmful treatments ('safety'). Candidate 'quality' criteria targeted common conditions where there are compelling indications for drug therapy based on UK and European guidelines [16-25]. Common conditions that may or may not require drug treatment for adequate management (depression, anxiety, dyspepsia, acute infections) or where we anticipated that undertreated patients would not be reliably identifiable from UK electronic data sets (chronic pain, chronic obstructive pulmonary disease, rheumatoid arthritis, thyroid disorders, epilepsy) were not considered. The following conditions were selected: primary and secondary prevention of vascular disease [26,27], management of diabetes [28], heart failure [29], atrial fibrillation [30], asthma [31] and osteoporosis [32,33].

In order to identify candidate safety criteria, the drug groups reported to be most frequently implicated in PDRM hospital admissions were identified from systematic reviews and large scale studies [1-4,34]. For each drug or drug group identified, a more extensive literature search was conducted in order to identify patient and/or treatment related risk factors that make patients particularly vulnerable to drug-related toxicity by virtue of age, medical history, co-prescription, treatment duration and/or dose. Standard medicines information resources [35-39] and the primary research literature were considered in addition to selected previously published medication assessment instruments [8-10,40]. Safety alerts in the British National Formulary [36], the UK National Prescribing Centre [38] and the Medicines and Healthcare products Regulatory Agency [39] were examined to identify prescribing that was less commonly reported to be implicated in drug-related hospital admissions but associated with severe harm. Candidate safety criteria targeting potentially harmful prescribing in vulnerable groups were identified drawing on the above literature sources (children and young adults, the elderly) as well as current clinical practice guidelines (heart failure [22]). Potentially important aspects of high-risk prescribing that relied on data items which are not consistently recorded in UK primary care electronic data sets (monitoring or achievement of international normalised ratio targets, monitoring of blood glucose in patients co-prescribed drugs known to enhance sensitivity to insulin or oral anti-diabetics, medication use in pregnancy/lactation) were excluded.

### RAND/UCLA Appropriateness Method (RAM) study

The RAND/University of California Los Angeles (UCLA) appropriateness method is a rigorous way of combining research evidence with expert opinion [41], and has previously been applied to develop explicit criteria for the assessment of a range of health care procedures including medication use [42]. A panel of ten members was selected with clinical, public health or academic expertise in medication use in UK primary care. The panel was composed of four general medical practitioners (of whom two had National Health Service prescribing improvement roles) and six pharmacists (including two academics with a special interest in primary care, two working in medicines governance at health board level, and two working directly with general practices). All ten participants completed two rounds of scoring.

The questionnaire aimed to classify candidate medication assessment criteria derived from the literature as either 'necessary' or 'appropriate' care (table 1). 'Necessary' is a more stringent rating standard than 'appropriate', because it represents care that would be '*improper' *not to be offered or avoided, whereas 'appropriate' is a more neutral balancing of net benefit or harm [43-45]. Following the RAM recommendations, ordinal scales of 1 to 9 were used for all ratings [43,46].

**Table 1:** Definitions of rating categories used in the modified RAM study [55]

Rating category	Definition
'Appropriate'	In an average patient, the expected health benefit usually exceeds the expected negative consequences by a sufficiently wide margin that prescribing is worthwhile, irrespective of cost
'Inappropriate'	In an average patient, the expected negative consequences usually exceed the expected health benefits by a sufficiently wide margin that prescribing is not worthwhile, irrespective of cost
'Necessary to do'	In an average patient, it would be considered improper care NOT to prescribe as stated, because (1) there is sufficient evidence, that the patient is likely to benefit AND (2) the likely benefit to the patient is large enough to be clinically significant
'Necessary to avoid'	In an average patient, it would be considered improper care to prescribe as stated, because (1) there is sufficient evidence, that the patient is likely to be harmed AND (2) the likely harm to the patient is large enough to be clinically significant

All candidate quality and safety assessment criteria were scored for 'appropriateness'. Candidate criteria with a median rating of 4 to 6 ('uncertain') or disagreement (three or more ratings of 7 to 9 *and *three or more ratings of 1 to 3) on the appropriateness scale were rejected. Those items with median ratings of 7 to 9 were accepted as 'appropriate' and those with median ratings of 1 to 3 as 'inappropriate'.

Candidate quality assessment criteria were additionally scored on a 'necessary to do' scale, where items with a median rating of 7 to 9 (= clearly necessary to do) were accepted. Candidate safety assessment criteria were additionally scored on a 'necessary to avoid' scale, where items with a median rating of 1 to 3 (= clearly necessary to avoid) were accepted. Candidate criteria with median ratings of < 7 on the 'necessary to do' and > 3 on the necessary to avoid scale and those showing disagreement (defined as above) were rejected. The concept of 'necessary to avoid' was an extension to the original RAM method to differentiate between prescribing that is 'generally not worthwhile' from 'improper' in safety terms (see box 2).

The ten RAM panel members were emailed the first round questionnaire and a summary of the supporting evidence base. Panellists were asked to rate each item with reference to an 'average' patient consulting an 'average' primary care clinician in 2009 based on both the evidence summary and their clinical judgement [44]. Panellists subsequently met for a full day, where a summary of the first round ratings was fed back to panellists anonymously. This formed the basis for a moderated discussion of each item before the second round ratings were placed. All findings reported in this paper are based on second round ratings.

### Delphi study

A random sample of general medical practitioners (GPs) and eligible pharmacists in Scotland and England was invited to participate by e-mail. In order to be eligible, pharmacists had to have experience of working in medicines governance, as a prescribing advisor or as a practice pharmacist. Twenty three (64%) GPs and 13 (36%) pharmacists agreed to participate.

The Delphi questionnaire listed the medication improvement topics to be scored together with a short summary of the scientific rationale for each topic. For each item, panellists were asked to state their level of agreement with the statement *'The described topic is a priority for collaborative quality improvement in primary care'*. The term 'collaborative' was used in order to emphasise that the intended purpose of this study was to identify priority topics for quality improvement rather than measures for judging practitioners or practices as part of performance management.

As in the RAM study, all ratings used an ordinal scale of 1 to 9 (1 = strongly disagree and 9 = strongly agree). Panellists were instructed to rate topics in relation to primary care in general, rather than their own practice. The first round ratings were summarised and returned to participants by email for a second round of scoring. Topics with second round median ratings of 7 to 9 without disagreement (30% or more ratings of 1 to 3 *and *30% or more ratings of 7 to 9) were accepted as 'priority', with median ratings of 8 or 9 defined as 'high priority'. All findings reported in this paper refer to second round ratings.

## Results

### Literature review and RAM study

The questionnaire listed 389 (100 quality and 289 safety) candidate assessment criteria. Upon completion of the second rating round, 318 (82%) candidates (93 quality and 225 safety) were accepted at the 'appropriate' and 275 (71%) items (73 quality and 202 safety) at the 'necessary' level. A number of candidate criteria were duplicates, in the sense that they were designed to determine thresholds beyond which care was judged appropriate and necessary. For example, 18 candidate quality assessment criteria related to glycated haemoglobin (HbA1c) levels beyond which treatment intensification was appropriate or necessary. Removing redundant candidate criteria yielded 52 quality and 124 safety assessment criteria to be included in the final set. Forty (77%) final quality assessment criteria and 107 (86%) final safety assessment criteria were derived from candidates accepted at the 'necessary' level. The results of the RAM study are summarised in tables 2 and 3 and the final list of quality and safety assessment criteria is presented in tables 4 and 5.

**Table 2:** Summary of final quality assessment criteria designed from candidates accepted in the modified RAM study as appropriate (App) or necessary to do (NecDo)

Condition targeted	Final quality assessment criteria				
	
	Medication quality category (MQ): Targeted prescribing	Associated PDRM event	Count		
			
			App	NecDo	Total
HYPERTENSION	MQ2: Selection of first line antihypertensives	CV events	-	1	13 (25%)
	MQ3: Blood pressure control	CV events	5	7	

DIABETES	MQ1: Use of ACEI/ARB if micro-albuminuria	DM complications	-	1	6 (12%)
	MQ2: Selection of metformin if overweight	DM complications	-	1	
	MQ3: HbA1c control	DM complications	2	2	

AT RISK OF PRIMARY/SECONDARY VASCULAR EVENTS	MQ1: Use of antiplatelet or anticoagulant	CV events	1	4	17 (33%)
	Use of statin	CV events	1	3	
	Use of ACEI or ARB in CHD	CV events	1	1	
	Use of beta blockers in CHD	CV events	-	2	
	MQ4: Achievement of target statin dose	CV events	-	4	

CHRONIC HEART FAILURE	MQ1: Use of ACEI or ARB	CHF progression	-	1	5(10%)
	Use of beta blocker	CHF progression	-	1	
	MQ2: Selection of beta blocker licensed for CHF	CHF progression	1	-	
	MQ4: Achievement of target ACEI/ARB dose	CHF progression	-	1	
	Achievement of target BB dose	CHF progression	-	1	

ATRIAL FIBRILLATION	MQ1: Use of antiplatelet or anticoagulant	Stroke/Embolism	-	3	4(8%)
	MQ2: Selection of warfarin in high risk of stroke	Stroke/Embolism	-	1	

ASTHMA	MQ1: Use of inhaled corticosteroid	Asthma exacerbation	1	3	4 (8%)

OSTEOPOROSIS	MQ1: Use of bone protecting agent	Fractures	-	1	3(6%)
	Use of calcium/vitamin D supplement	Fractures	-	2	

**Total**			**12**	**40**	**52 (100%)**

**Medication Quality categories**					
1. INDICATION	MQ1: Need for treatment to control condition		4	22	26 (52%)

2. SELECTION	MQ2: Selection of first line option within drug class		1	3	4 (8%)

3. INTENSITY	MQ3: Achievement of intermediate outcome target		7	9	16 (31%)
	MQ4: Achievement of target dose		-	6	6 (12%)

**Criteria restricted to the elderly**					
Aged ≥ 75			4	4	8 (15%)
Aged ≥ 80			-	2	2 (4%)

MQ1 to 4 refer to medication quality categories as specified in the bottom half of the table.

**Table 3:** Summary of final safety assessment criteria designed from candidates accepted in the modified RAM study as inappropriate (InApp) or necessary to avoid (NecAv)

High-risk drug/patient group	Final safety assessment criteria				
	
	Medication safety category (MS): Targeted prescribing/monitoring	Associated PDRM event	Count		
			InApp	NecAv	Total
**Drugs frequently implicated in PDRM hospital admissions**					

ANTIPLATELET	MS1: Use without gastroprotection	GI toxicity/bleeding	1	4	5 (4%)

DIURETIC	MS1: Unmet need for allopurinol in thiazide users	Gout	-	1	11 (9%)
	MS3: Use of thiazides in CKD	Renal toxicity	-	1	
	Use of aldosterone antagonist in CKD	Hyperkalaemia	-	1	
	MS6: Excess duration of potassium supplement	Hyperkalaemia	-	1	
	MS8: Inconsistent monitoring of U&E's	Electrolyte disturbances	2	5	

NSAID	MS1: Unmet need for gastroprotection	GI toxicity/bleeding	1	4	18 (15%)
	MS2: Use of COX II selective agents in aspirin users	CV events	-	1	
	Paracetamol not tried first	General NSAID toxicity	1	2	
	MS3: Use in CKD	Renal toxicity	-	2	
	Use of COX II selective agents in high CV risk	CV events	-	2	
	MS4: Co-prescription with diuretic and/or ACEI or ARB	Renal toxicity	2	3	

ANTICOAGULANT	MS2: Use of warfarin in AF and low risk of stroke	Bleeding	-	1	11 (9%)
	MS4: Co-prescription of high-risk anti-infectives	Bleeding	1	9	

OPIOID	MS1: No laxative co-prescribed in strong opioid users	Constipation	1	1	2 (2%

BETA BLOCKER	MS3: Use in asthma	Asthma exacerbation	1	2	4 (3%)
	MS4: Co-prescription with verapamil/diltiazem	Bradycardia	-	1	

ACEI/ARB	MS8: Inconsistent monitoring of U&E's	Hyperkalaemia	-	2	2 (2%)

ANTIDIABETIC	MS3: Use of long acting sulphonylureas in CKD	Hypoglycaemia	-	1	2 (2%)
	Use of metformin in CKD	Lactic acidosis	-	1	

DIGOXIN	MS5: Excessive dose in CKD or the elderly	Digoxin toxicity	-	2	10 (8%)
	Excessive dose in patients on interacting drugs		-	6	
	MS8: Inconsistent monitoring of U&E's		-	2	

ORAL STEROID	MS1: Unmet need for bone protecting agents	Bone fracture	-	2	2 (2%)

**Other drugs implicated in severe adverse drug events**					
DMARD	MS7: Lack of dose instructions/Use of 2 strengths	Miscellaneous	-	2	10 (8%)
	MS8: Inconsistent monitoring of FBC	Blood dyscrasias	2	6	
FEMALE	MS3: Use of estrogens in women w/o hysterectomy	Gynaecological cancer	-	1	7 (6%)
STEROIDS	MS3: Use in women with CVD or CVD risk > 20%	Vascular events	2	2	
	MS6: Excess duration in postmenopausal women		1	1	
AMIODARONE	MS8: Inconsistent monitoring of thyroid function	Thyroid disturbances	-	1	1 (1%)
THEOPHYLLIN	MS2: Use without inhaled anticholinergics/steroids	Theophylline toxicity	-	1	1 (1%)
STATIN	MS5: Excessive dose in patients on interacting drugs	Rhabdomyolysis	-	5	5 (4%)

**Particularly vulnerable patient groups**					
ELDERLY	MS3: Miscellaneous drugs to be avoided	Miscellaneous	1	10	18 (15%)
	MS6: Miscellaneous drugs for excessive duration	Miscellaneous	-	7	
HEART FAILURE	MS3: Miscellaneous drugs to be generally avoided	HF exacerbation	-	10	10 (8%)
CHILDREN	MS3: Miscellaneous drugs to be generally avoided	Miscellaneous	1	4	5 (4%)

**Total**			**17**	**107**	**124 (100%)**

**Medication Safety (MS) categories**					

1. INDICATION	MS1: Unmet need for risk mitigating drug		3	12	15 (12%)

2. SELECTION	MS2: High risk drug without compelling indication		1	5	64 (52%)
	MS3: Drug-disease or Drug-age interaction		5	37	
	MS4: Drug-Drug interaction (DDI)		3	13	

3. INTENSITY	MS5: Excessive dose		-	13	23 (19%)
	MS6: Excessive duration		1	9	

4. COMPLIANCE	MS7: Issues related to patient compliance		-	2	2 (2%)

5. MONITORING	MS8: Inconsistent laboratory monitoring		4	16	20 (16%)

**Criteria restricted to the elderly**					
Aged ≥ 65			3	21	24 (19%)
Aged ≥ 75			2	9	11 (9%)
Aged ≥ 85			1	-	1 (1%)

MS 1 to 8 refer to medication quality categories as specified in the bottom half of the table.

**Table 4:** Quality assessment criteria generated from candidates that the RAM panel classified as 'appropriate but not necessary to do' (A) and 'appropriate and necessary to do' (N)

Topic No.	Treatment targeted - Associated PDRM event (Medication quality category)
HYPERTENSION	

**Q1**	**Selection of first line antihypertensives - Hypertension complications (MQ2)**
	1. (N) Patient with HTN and without CHD - is started on antihypertensive treatment with a first-line antihypertensive
**Q2**	**Treatment to blood pressure (BP) target - Hypertension complications (MQ3)**
	Patient aged < 75 years, who has a history of hypertension WITHOUT complications
	2. (N) and BP is > 150/90 mmHg on < 3 antihypertensive drugs - has antihypertensive treatment intensified
	3. (N) and BP is > 140/85 mmHg on < 2 antihypertensive drugs - has antihypertensive treatment intensified
	4. (A) and BP is > 140/85 mmHg on < 3 antihypertensive drugs - has antihypertensive treatment intensified
	Patient aged ≥ 75 years, who has a history of hypertension WITHOUT complications
	5. (N) and BP is > 150/90 mmHg on < 2 antihypertensive drugs - has antihypertensive treatment intensified
	6. (N) and BP is > 140/85 mmHg without antihypertensive treatment - has antihypertensive treatment started
	7. (A) and BP is > 150/90 mmHg on < 3 antihypertensive drugs - has antihypertensive treatment intensified
	8. (A) and BP is > 140/85 mmHg on < 2 antihypertensive drugs - has antihypertensive treatment intensified
	Patient aged < 75 years, who has a history of hypertension WITH complications
	9. (N) and BP is > 130/80 mmHg on < 2 antihypertensive drugs - has antihypertensive treatment intensified
	Patient aged ≥ 75 years, who has a history of hypertension WITH complications
	10. (N) and BP is > 140/85 mmHg on < 2 antihypertensive drugs - has antihypertensive treatment intensified
	11. (N) and BP is > 130/80 mmHg without antihypertensive treatment - has antihypertensive treatment intensified
	12. (A) and BP is > 140/85 mmHg on < 3 antihypertensive drugs - has antihypertensive treatment intensified
	13. (A) and BP is > 130/80 mmHg on < 2 antihypertensive drugs - has antihypertensive treatment intensified

DIABETES MELLITUS	

**Q3**	**Treatment to HbA1c target - Diabetes complications (MQ3)**
	Patient with diabetes mellitus type 2,
	14. (N) who has HbA1c of > 7% on < 2 oral antidiabetic drugs - has antidiabetic treatment intensified
	15. (N) who has HbA1c of > 9% on < 3 oral antidiabetic drugs - has antidiabetic treatment intensified
	16. (A) who has HbA1c of 6.6 to 7% without antidiabetic treatment - has antidiabetic treatment intensified
	17. (A) who has HbA1c of 7.6 to 9% on < 3 oral antidiabetic drugs - has antidiabetic treatment intensified
**Q4**	**Selection of first line oral antidiabetic - Diabetes complications (MQ2)**
	18. (N) Patient with diabetes mellitus type 2, who is overweight - is started on metformin
**Q5**	**Indication for ACEI or ARB in patients with renal complications - Diabetes complications (MQ1)**
	19. (N) Patient with diabetes mellitus and micro-albuminuria - is prescribed an ACEI or ARB

AT RISK OF/MANIFEST VASCULAR DISEASE	

**Q6**	**Indication for statin in patients with manifest vascular disease or risk factors - Vascular events (MQ1)**
	20. (N) Patient with previous vascular events (MI, stroke or TIA) - is prescribed a statin
	21. (N) Patient with peripheral vascular disease - is prescribed a statin
	22. (N) Patient aged > 40 with DM without established vascular disease - is prescribed a statin
	23. (A) Patient with 10 year CVD risk > 20% without diabetes - is prescribed a statin
**Q7**	**Treatment to target statin dose in patients with manifest vascular disease or risk factors - Vascular events (MQ4)**
	24. (N) Patient with previous vascular events (MI, stroke or TIA) - is prescribed simvastatin ≥ 40 mg/d (or equivalent)
	25. (N) Patient with peripheral vascular disease - is prescribed simvastatin ≥ 40 mg/d (or equivalent)
	26. (N) Patient aged > 40 with DM without established vascular disease - is prescribed simvastatin ≥ 40 mg/d (or equiv.)
	27. (N) Patient with 10 year CVD risk > 20% without diabetes - is prescribed simvastatin ≥ 40 mg/d (or equivalent)
**Q8**	**Indication for thrombo-embolic prophylaxis in patients with CHD - Vascular events (MQ1)**
	28. (N) Patient with previous vascular events (MI, stroke or TIA) - is prescribed any thrombo-embolic prophylaxis
	29. (N) Patient with a history of peripheral vascular disease - is prescribed any thrombo-embolic prophylaxis
	Indication for dual antiplatelets in CHD with a history of ACS - Vascular events (MQ1)
	30. (N) Patient with previous stroke/TIA - is co-prescribed aspirin and dipyridamole (unless on warfarin or clopidogrel)
	31. (N) Patient with ACS 0 to 3 months ago - is co-prescribed aspirin and clopidogrel (unless on warfarin)
	32. (A) Patient with ACS 4 to 9 months ago - is co-prescribed aspirin and clopidogrel (unless on warfarin)
**Q9**	**Indication for beta blockers in CHD - Vascular events (MQ1)**
	33. (N) Patient with a history of acute coronary syndrome - is prescribed a beta blocker
	34. (N) Patient with stable angina without a history of acute coronary syndrome - is prescribed a beta blocker
**Q10**	**Indication for ACEI or ARB in CHD - Vascular events (MQ1)**
	35. (N) Patient with a history of acute coronary syndrome - is prescribed an ACEI or ARB
	36. (A) Patient with stable angina without a history of acute coronary syndrome - is prescribed an ACEI or ARB

CHRONIC HEART FAILURE	

**Q11**	**Indication for ACEI or ARB in CHF - Heart failure progression (MQ1)**
	37. (N) Patient with CHF - is prescribed an ACE or ARB
**Q12**	**Indication for Beta blocker in CHF - Heart failure progression (MQ1)**
	38. (N) Patient with CHF - is prescribed a beta blocker
	**Selection of licensed beta blocker in CHF - Heart failure progression (MQ2)**
	39. (A) Patient with CHF and treated with a BB - is prescribed a BB licensed for CHF
**Q13**	**Treatment to target dose (ACEI and ARB) in CHF - Heart failure progression (MQ4)**
	40. (N) Patient with CHF and treated with an ACEI or ARB - has achieved the recommended target dose
**Q13**	**Treatment to target dose (beta blocker) - CHF- Prevention of heart failure progression (MQ4)**
	41. (N) Patient with CHF and treated with a beta blocker - has achieved the recommended target dose

ATRIAL FIBRILLATION	

**Q14**	**Indication for thrombo-embolic prophylaxis in AF - Thrombo-embolism (MQ1)**
	42. (N) Patient with atrial fibrillation and a CHADS2 score of 0 or 1 - is prescribed thrombo-embolic prophylaxis
	43. (N) Patient with atrial fibrillation and a CHADS2 score of 2 - is prescribed thrombo-embolic prophylaxis
	44. (N) Patient with atrial fibrillation and a CHADS2 score ≥ 3 - is prescribed thrombo-embolic prophylaxis
**Q15**	**Selection of thrombo-embolic prophylaxis in AF - Thrombo-embolism (MQ2)**
	45. (N) Patient with AF and a CHADS_2 _score ≥ 3 treated with an antithrombotic - is prescribed an oral anticoagulant

ASTHMA	

**Q16**	**Indication for inhaled corticosteroids in asthma - Asthma exacerbation (MQ1)**
	Patient aged > 4 with asthma but without COPD and
	46. (N) is treated with a step 3 drug* - is also prescribed an inhaled corticosteroid
	47. (N) has received oral prednisolone in last 12 weeks - is also prescribed an inhaled corticosteroid
	48. (N) has received ≥ 3 prescriptions of SABAs in last 12 weeks - is also prescribed an inhaled corticosteroid
	49. (A) has received 2 prescriptions of SABAs in last 12 weeks - is also prescribed an inhaled corticosteroid
	* long acting beta agonist, leukotriene receptor antagonist or theophylline

OSTEOPOROSIS	

**Q17**	**Indication for bone protecting agents in patients with osteoporosis - Fractures (MQ1)**
	50. (N) Female patient with osteoporosis who had a vertebral fracture - is prescribed a bone protecting agent*
	* a bisphosphonate, strontium ranelate, raloxifene or teriparatide
**Q18**	**Indication for Calcium/vitamin D in patients at risk of osteoporosis - Fractures (MQ1)**
	51. (N) Female patient aged ≥ 80 who is housebound - is prescribed calcium and vitamin D
	52. (N) Female patient aged ≥ 80 who lives in a nursing home/residential care - is prescribed calcium and vitamin D

The criteria are organised hierarchically by medical condition, followed by the drug group targeted, quality topic scored in the Delphi study (Q) and by medication use quality category (MQ). MQ1 = indication for beneficial treatment, MQ2 = Selection of most effective option within drug class, MQ3 = Achievement of intermediate outcome target, MQ4 = Achievement of target dose

**Table 5:** Safety assessment criteria generated from candidates that the RAM panel classified as 'inappropriate' (I) or 'necessary to avoid' (N)

**Topic No**.	Treatment targeted - Associated PDRM event (Medication safety category)
**A. DRUGS FREQUENTLY IMPLICATED IN PDRM HOSPITAL ADMISSIONS**	

ANTIPLATELETS	

**S1**	**High-risk use without gastro-intestinal protection (GIP) - GI toxicity/bleeding (MS1)**
	1. (N) Patient with previous peptic ulcer (PU) treated with low dose aspirin - is not prescribed GIP
	2. (N) Patient aged ≥ 65 treated with warfarin AND low dose aspirin - is not prescribed GIP
	3. (N) Patient aged ≥ 65 treated with warfarin AND clopidogrel - is not prescribed GIP
	4. (N) Patient aged ≥ 65 treated with low dose aspirin AND clopidogrel - is not prescribed GIP
	5. (I) Patient aged ≥ 75 years treated with low dose aspirin - is not prescribed GIP

NSAIDS	

**S1**	**High-risk use without gastroprotection (GIP) - GI toxicity/bleeding (MS1)**
	6. (N) Patient with previous PU treated with an oral NS NSAID for > 12 weeks - is not prescribed GIP
	7. (N) Patient is aged ≥ 75 years treated with an oral NS NSAID for > 12 weeks - is not prescribed GIP
	8. (I) Patient is aged 65 to 74 treated with an oral NS NSAID for > 12 weeks - is not prescribed GIP
**S1**	**High-risk use without gastroprotection - GI toxicity/bleeding (MS1)**
	9. (N) Patient aged ≥ 65 treated with warfarin AND an oral NS NSAID - is not prescribed GIP
	10. (N) Patient aged ≥ 65 treated with low dose aspirin AND an oral NS NSAID for > 12 weeks - is not prescribed GIP
**S2**	**High risk drug without compelling indication - General drug specific toxicity (MS2)**
	11. (N) Patient aged ≥ 65 - is prescribed an oral NSAID for osteoarthritis without previous trial of full dose paracetamol
	12. (N) Patient aged ≥ 75 - is prescribed an oral NSAID for minor trauma without previous trial of full dose paracetamol
	13. (I) Patient aged 65 to 74 - is prescribed an oral NSAID for minor trauma without previous trial of full dose paracetamol
**S3**	**High-risk selection in renal impairment - Renal toxicity (MS3)**
	14. (N) Patient with CKD stage 3 - is prescribed an oral NSAID
	15. (N) Patient with CKD stage 4 or 5 - is prescribed an oral NSAID
**S3**	**Drug-Drug interaction (additive toxicity) - Renal toxicity (MS4)**
	16. (N) Patient aged ≥ 65 treated with an ACEI or ARB but no diuretic - is co-prescribed an oral NSAID
	17. (N) Patient aged ≥ 75 treated with a diuretic but no ACEI or ARB - is co-prescribed an oral NSAID
	18. (N) Patient treated with an ACEI or ARB AND a diuretic - is co-prescribed an oral NSAID
	19. (I) Patient aged ≤ 65 treated with an ACEI or ARB but no diuretic - is co-prescribed an oral NSAID
	20. (I) Patient aged 65 to 74 treated with a diuretic but no ACEI or ARB - is co-prescribed an oral NSAID
**S4**	**High-risk drug without compelling indication - CV events (MS2)**
	21. (N) Patient treated with low-dose aspirin - is prescribed an oral COX II selective NSAID
**S5**	**High-risk selection in patients at high vascular risk- Vascular events (MS3)**
	22. (N) Patient aged > 40 and CVD risk > 20% - is prescribed a COX II selective NSAID
	23. (N) Patient with a history of vascular events - is prescribed a COX II selective NSAID

DIURETICS	

**S6**	**Monitoring of U&E's - Electrolyte imbalance (MS8)**
	24. (N) Patient treated with a potassium sparing diuretic -had no U&Es check before treatment start
	25. (N) Patient treated with a potassium sparing diuretic - had no U&Es check in the last 48 weeks
	26. (N) Patient treated with a loop diuretic - had no U&Es check before treatment start
	27. (N) Patient treated with a loop AND a thiazide diuretic or metolazone - had no U&Es check in the last 24 weeks
	28. (N) Patient treated with a potassium sparing diuretic AND an ACEI or ARB - had no U&Es check in the last 48 weeks
	29. (I) Patient treated with a potassium wasting diuretic - had no U&Es check in the last 48 weeks
	30. (I) Patient treated with a potassium sparing diuretic AND an ACEI or ARB - had no U&Es check in the last 24 weeks
**S7**	**High-risk selection in renal impairment - Renal toxicity/Treatment failure (MS3)**
	31. (N) Patient with chronic kidney disease stage 4 or 5 - is prescribed a thiazide diuretic
**S8**	**High-risk use without allopurinol - Gout (MS1)**
	32. (N) Patient with a history of gout and treated with a thiazide diuretic - is not prescribed allopurinol
**S9**	**High-risk selection in renal impairment - Electrolyte imbalance (MS3)**
	33. (N) Patient with CKD stage 4 or 5 - is prescribed an aldosterone antagonist
**S10**	**Excess duration - Electrolyte imbalance (MS6)**
	34. (N) Patient treated with a potassium (KI) sparing diuretic - is prescribed a K+ supplement for ≥ 4 weeks

ANTICOAGULANTS	

**S11**	**Drug-Drug interaction (pharmacokinetic) - Bleeding (MS4)**
	35. (N) Patient treated with warfarin - is co-presribed a macrolide
	36. (N) Patient treated with warfarin - is co-prescribed a sulfonamide
	37. (N) Patient treated with warfarin - is co-prescribed an azole antifungal
	38. (N) Patient treated with warfarin - is co-prescribed metronidazole
	39. (N) Patient treated with warfarin - is co-prescribed chloramphenicol
	40. (N) Patient treated with warfarin- is co-prescribed isoniazid
	41. (N) Patient treated with warfarin - is co-prescribed rifampin
	42. (N) Patient treated with warfarin - is co-prescribed griseofulvin
	43. (N) Patient treated with warfarin - is co-prescribed ribavirin
	44. (I) Patient treated with warfarin - is co-prescribed tetracyclines
**S12**	**High risk drug without compelling indication- Bleeding (MS2)**
	45. (N) Patient with atrial fibrillation - is prescribed warfarin despite CHADS2 score = 0

OPIOIDS - CONSTIPATION	

**S13**	**High-risk use without laxative - Constipation (MS1)**
	46. (N) Patient treated with a strong opioid (morphine > 10 mg or equivalent) for > 4 weeks - is not prescribed a laxative
	47. (I) Patient aged ≥ 65 treated with a strong opioid (morphine > 10 mg or equivalent) - is not prescribed a laxative

BETA BLOCKERS	

**S14**	**Drug-drug interaction (additive toxicity) - Bradycardia (MS4)**
	48. (N) Patient treated with a beta-blocker - is co-prescribed verapamil or diltiazem
**S15**	**High-risk selection in asthma - Asthma exacerbation (MS3)**
	49. (N) Patient with active asthma (prescribed beta agonist inhaler in last year) without COPD - is prescribed any oral BB
	50. (N) Patient with active asthma without COPD - is prescribed a non-cardio-selective oral BB
	51. (I) Patient with active asthma without COPD - is prescribed beta-blocker eye drops

ACE INHIBITORS (ACEIs) AND ANGIOTENSIN RECEPTOR BLOCKERS (ARBs)	

**S6**	**Monitoring of U&E's - Electrolyte imbalance (MS8)**
	52. (N) Patient co-prescribed an ACEI AND ARB - has not had a U&Es check > 24 weeks ago
	Monitoring of U&E's - Electrolyte imbalance (MS8)
	53. (N) Patient prescribed an ACEI or ARB - has not had a U&Es check before treatment start

ANTIDIABETICS	

**S16**	**High-risk selection in renal impairment- Lactic acidosis (MS3)**
	54. (N) Patient with chronic kidney disease (CKD) stage 4 or 5 - is prescribed metformin
**S17**	**High-risk selection in renal impairment - Hypoglycaemia (MS3)**
	55. (N) Patient with CKD stage 4 or 5 - is prescribed a sulphonylurea other than gliclazide or tolbutamide

DIGOXIN	

**S18**	**Excessive dose (Elderly) - General digoxin toxicity (MS5)**
	56. (N) Patient aged ≥ 65 years - is prescribed digoxin ≥ 250 mcg/day
	57. (N) Patient with CKD stage 3, 4 or 5 (eGFR < 60) - is prescribed digoxin ≥ 250 mcg/day
**S18**	**Excessive dose (DDI without dose adjustment) - General digoxin toxicity (MS5)**
	58. (N) Patient treated with digoxin and amiodarone - is prescribed digoxin ≥ 250 mcg/day
	59. (N) Patient treated with digoxin and propafenone - is prescribed digoxin ≥ 250 mcg/day
	60. (N) Patient treated with digoxin and chloroquine or hydroxychloroquine - is prescribed digoxin ≥ 250 mcg/day
	61. (N) Patient treated with digoxin and quinine - is prescribed digoxin ≥ 250 mcg/day
	62. (N) Patient treated with digoxin and a calcium channel blocker * - is prescribed digoxin ≥ 250 mcg/day
	63. (N) Patient treated with digoxin and ciclosporin - is prescribed digoxin ≥ 250 mcg/day
	* lercanidipine, nicardipine, nifedipine, diltiazem, verapamil)
**S8**	**Monitoring of U&E's - General digoxin toxicity (MS8)**
	64. (N) Patient is co-prescribed a potassium wasting diuretic AND digoxin with last U&Es check before treatment start
	65. (N) Patient is co-prescribed a potassium wasting diuretic AND digoxin with last U&Es check > 48 weeks ago

CORTICOSTEROIDS	

**S19**	**High-risk use without bone protecting agent - Bone fracture (MS1)**
	66. (N) Patient aged ≥ 65 years treated with an oral corticosteroid for ≥ 12 weeks - is not prescribed bone protection *
	67. (N) Patient with low trauma fracture and treated with an oral corticosteroid for ≥ 12 weeks - is not prescribed bone protection*
	*a bisphosphonate, calcitriol or hormone replacement therapy

**B. OTHER HIGH RISK DRUGS**	

DMARDS	

**S20**	**High-risk drug without taking action to ensure patient compliance - General toxicity (MS7)**
	68. (N) Patient treated with methotrexate - has not been given explicit dose instructions of weekly dosing
	69. (N) Patient treated with methotrexate - is prescribed > 1 strength of methotrexate tablets
**S21**	**Monitoring of full blood count (FBC) - Blood dyscrasias (MS8)**
	70. (N) Patient treated with auranofin - had no FBC check in the last 8 weeks
	71. (N) Patient treated with aurothiomalate - had no FBC check in the last 8 weeks
	72. (N) Patient treated with penicillamine - had no FBC check in the last 8 weeks
	73. (N) Patient treated with leflunomide - had no FBC check in the last 12 weeks
	74. (N) Patient treated with methotrexate - had no FBC check in the last 12 weeks
	75. (N) Patient treated with azathioprine - had no FBC check in the last 12 weeks
	76. (I) Patient treated with cyclophosphamide - had no FBC check in the last 24 weeks
	77. (I) Patient treated with sulfasalazine - had no FBC check in the last 24 weeks

FEMALE HORMONES	

**S22**	**Selection in patients at high vascular risk - Vascular events (MS3)**
	78. (N) Patient with previous vascular disease/events - is prescribed any hormone replacement therapy (HRT)
	79. (N) Patient with an estimated 10 year CVD risk ≥ 20% - is prescribed combined contraceptives
	80. (I) Patient with an estimated 10 year CVD risk ≥ 20% and aged 50 to 59 - is prescribed combined HRT
	81. (I) Patient with an estimated 10 year CVD risk ≥ 20% and aged ≥ 60 - is prescribed (any) HRT
**S23**	**Excess duration - Gynaecological cancer (MS6)**
	82. (N) Patient aged ≥ 50 - is prescribed combined HRT for ≥ 5 years
	83. (N) Patient aged ≥ 50 without hysterectomy - is prescribed estrogens without cyclical progestogen
	84. (I) Patient aged ≥ 50 - is prescribed estrogens only HRT for ≥ 5 years

AMIODARONE	

**S24**	**Monitoring of thyroid function - Hypo-/Hyperthyroidism (MS8)**
	85. (N) Patient prescribed amiodarone - had no thyroid function test in last 9 months

THEOPHYLLINE	

**S25**	**High-risk drug without compelling indication - General theophylline toxicity (MS2)**
	86. (N) Patient aged ≥ 65 with COPD - is prescribed theophylline without use of a long acting beta2 - agonist or antimuscarinic

STATINS	

**S26**	**Excessive dose (DDI without dose adjustment) - Rhabdomyolysis (MS5)**
	87. (N) Patient treated with simvastatin and an HIV protease inhibitor - is prescribed simvastatin > 10 mg/day
	88. (N) Patient treated with simvastatin and ciclosporin - is prescribed simvastatin > 10 mg/day
	89. (N) Patient treated with simvastatin and verapamil - is prescribed simvastatin > 10 mg/day
	90. (N) Patient treated with simvastatin and a fibrate (except fenofibrate) - is prescribed simvastatin > 10 mg/day
	91. (N) Patient treated with simvastatin and amiodarone - is prescribed simvastatin > 20 mg/day

**C. PATIENT GROUPS PARTICULARLY VULNERABLE TO ADVERSE DRUG EVENTS**	

ELDERLY PATIENTS	

**S27**	**High-risk drug selection in the elderly - Miscellaneous (MS3)**
	92. (N) Patient aged ≥ 65 with dementia - is prescribed a TCA
	93. (N) Patient aged ≥ 65 with dementia but no psychosis - is prescribed an antipsychotic
	94. (N) Patient aged ≥ 65 with dementia and psychosis - is prescribed antipsychotic other than risperidone
	95. (N) Patient aged ≥ 65 - is prescribed a long acting benzodiazepine
	96. (N) Patient aged ≥ 65 with Parkinson's disease - is prescribed an antipsychotic other than quetiapine or clozapine
	97. (N) Patient aged ≥ 65 with Parkinson's disease - is prescribed a phenothiazine antiemetic
	98. (N) Patient aged ≥ 75 - is prescribed a TCA
	99. (N) Patient aged ≥ 75 - is prescribed a short acting benzodiazepine
	100. (N) Patient aged ≥ 75 - is prescribed a Z-drug
	101. (N) Patient aged ≥ 75 - is prescribed an antihistamine with antimuscarinic properties
	102. (A) Patient aged ≥ 85 - is prescribed an antispasmodic with antimuscarinic properties
**S27**	**Excess duration - Miscellaneous (MS6)**
	103. (N) Patient aged ≥ 65 - is prescribed a TCA for ≥ 4 weeks
	104. (N) Patient aged ≥ 65 - is prescribed a short acting benzodiazepine for ≥ 4 weeks
	105. (N) Patient aged ≥ 65 - is prescribed a Z-drug for ≥ 4 weeks
	106. (N) Patient aged ≥ 65 - is prescribed an antispasmodic with antimuscarinic properties for ≥ 4 weeks
	107. (N) Patient aged ≥ 65 with dementia and psychosis - is prescribed risperidone for ≥ 12 weeks
	108. (N) Patient aged 66 to 75 - is prescribed an antihistamine with antimuscarinic properties for ≥ 4 weeks
	109. (N) Patient aged ≥ 75 - is prescribed urologicals with antimuscarinic properties for ≥ 4 weeks

PATIENTS WITH HEART FAILURE	

**S28**	**Use in heart failure - Heart failure exacerbation (MS3)**
	110. (N) Patient with chronic heart failure - is prescribed a class 1 or 3 antiarrhythmics except amiodarone
	111. (N) Patient with chronic heart failure - is prescribed verapamil or diltiazem
	112. (N) Patient with chronic heart failure - is prescribed minoxidil
	113. (N) Patient with chronic heart failure - is prescribed any oral NSAID
	114. (N) Patient with chronic heart failure - is prescribed a glitazone
	115. (N) Patient with chronic heart failure - is prescribed a tricyclic antidepressant
	116. (N) Patient with chronic heart failure - is prescribed itraconazole
	117. (N) Patient with chronic heart failure - is prescribed other antifungals (e.g. ketoconazole, fluconazole)
	118. (N) Patient with chronic heart failure - is prescribed tadalafil
	119. (N) Patient with chronic heart failure - is prescribed disulfiram

CHILDREN AND YOUNG ADULTS	

**S29**	**Use in children - Miscellaneous (MS3)**
	120. (N) Patient aged ≤ 20 - is prescribed a phenothiazine anti-emetic
	121. (N) Patient aged ≤ 16 who has no record of Kawasaki disease - is prescribed aspirin
	122. (N) Patient aged ≤ 12 - is prescribed a tetracycline
	123. (N) Patient aged ≤ 18 - is prescribed an antidepressant other than fluoxetine
	124. (I) Patient aged ≤ 18 - is prescribed fluoxetine

The criteria target high-risk use of (A) drugs frequently implicated in PDRM hospital admissions, (B) other drugs implicated in severe PDRM events and (C) medication use in vulnerable groups. Within each domain A to C, the criteria are organised hierarchically by the high-risk drug (group) that is the focus of each criterion, followed by safety topic scored in the Delphi study (S) and medication use safety category (MS). MS1 = Indication for risk mitigating drug; MS2 = High risk drug without compelling indication; MS3 = Drug-disease or Drug-age interaction; MS4 = Drug-Drug interaction (DDI); MS5 = Excessive dose; MS6 = Excessive duration; MS7 = Prescribing issues linked to patient compliance; MS8 = Inconsistent monitoring

Table 2 shows the number of accepted quality assessment criteria categorised (1) by medical condition and (2) by four medication quality categories (MQ 1 to 4) referring to 'need (indication)', 'selection' or 'intensity' of drug treatment that were informed by available taxonomies [13-15]. The majority (87%) of the final 52 quality assessment criteria focus on the prevention (including diabetes mellitus) or management of vascular disease with lower proportions addressing asthma (8%) and osteoporosis (6%). Over half (52%) of final quality criteria target patients with unmet indications for drug therapy (MQ1) and 43% focus on treatment intensity (MQ3 and MQ4) for effective disease management with the remainder (8%) targeting selection of first line agents within a therapeutic class (MQ2).

Similarly, table 3 categorises the number of accepted safety assessment criteria generated (1) by high-risk drug or patient group targeted and (2) by eight medication safety categories (MS 1 to 8), referring to 'need (indication)', 'selection', treatment 'intensity', 'compliance' issues and 'monitoring'.

The majority of safety assessment criteria are drug-focussed (74%), either targeting drugs reported to be frequently implicated in PDRM hospital admissions (54% - section A) or others implicated in severe preventable harm (20% - section B). The remainder (26% - section C) target medication use in particularly vulnerable groups, namely the elderly (15%), patients with heart failure (8%) and children (4%). Over a third (36%) of final safety assessment criteria focus on potentially harmful use of NSAIDs, antiplatelets, anticoagulants and diuretics, the drug groups most frequently implicated in PDRM hospital admissions [1].

Over half (52%) of safety criteria target the selection of high-risk drugs (MS2 to 4), either for indications where safer (and equally effective) alternatives exist (MS2) or in patients particularly susceptible to adverse reactions because of age/co-morbidity (MS3) or co-prescription (MS4). A further 15 (12%) criteria target omissions of drugs indicated to mitigate the risk of adverse events from high-risk treatments (MS1), while twenty (16%) criteria target inconsistent laboratory monitoring (MS8). Two (2%) criteria focus on prescribing that may jeopardise patient compliance with methotrexate dosing schedules (MS7).

The majority of quality (81%) and safety (71%) assessment criteria are not restricted to the elderly (patients aged 65 years or older).

### Delphi study

Grouping of thematically related assessment criteria that were derived from candidates accepted at the 'necessary' level yielded a total of 47 (18 quality and 29 safety) medication improvement topics to be rated by the Delphi panel. Thirty-six Delphi study participants completed a first round and 26 (73%) a second round questionnaire (table 6). Fifteen (83%) quality and 23 (79%) safety topics were accepted as 'priorities for quality improvement in primary care'. Eleven (7 quality and 4 safety) topics were classified as 'high priorities' and nine (3 quality and 6 safety) topics were rejected because of lower than stipulated median ratings (table 7). There were no differences between pharmacists and GPs with respect to the assignment of priority levels, and no panel disagreement on any topic (defined in terms of variation in scoring as detailed in the methods section above).

**Table 6:** Delphi study: Demographics of the 26 panellists, who completed both rounds of ratings

	Pharmacists n = 9 (35%)		General practitioners n = 17 (65%)		Total
	
	*Currently*	*Previously*	*Currently*	*Previously*	
Works in primary care	7	2	17	-	26 (100%)
Has a prescribing role	2	1	17	-	20 (77%)
Has a strategic role	2	-	1	1	4 (15%)
Mean age in years (SD)	47 (9)		47 (9)		47 (9)
Mean years since training completed (SD)	22 (11)		22 (9)		23 (10)
Mean years of experience of working in primary care (SD)	11 (11)		19 (8)		15 (8)

**Table 7:** Delphi study priority ratings by the 26 panellists

Topic		*Median*	*Mean*	*Priority *
	**Accepted as priorities**			

	***Quality***			
Q 16	Not using inhaled corticosteroids in patients with uncontrolled asthma	8	8.0	++
Q 15	Not using oral anticoagulants in patients with AF and high risk of stroke	8	7.9	++
Q 11	Not using ACEIs or ARBs in patients with a history of chronic heart failure	8	7.9	++
Q 14	Not using thrombo-embolic prophylaxis in AF patients at low/moderate risk of stroke	8	7.7	++
Q 5	Not using ACEIs or ARBs in patients with DM and renal complications	8	7.7	++
Q 12	Not using beta blockers in patients with a history of chronic heart failure	8	7.7	++
Q 4	Not using metformin as first line antidiabetic in overweight type 2 diabetics	8	7.6	++
Q 8	Not using antiplatelets in patients at risk of vascular events	7	7.5	+
Q 6	Not using statins in patients at high risk of cardiovascular events	7	7.4	+
Q 17	Not using bone sparing agents in female patients at high risk of fractures	7	7.3	+
Q 3	Low intensity antidiabetic treatment despite suboptimal HbA1c control	7	7.2	+
Q 10	Not using ACEIs or ARBs in patients with a history of ACS	7	7.0	+
Q 2	Low intensity antihypertensive treatment despite suboptimal BP control	7	6.9	+
Q 9	Not using beta blockers in coronary heart disease	7	6.8	+
Q 7	Underdosing of statins in patients at high risk of cardiovascular events	7	6.7	+
	***Safety***			
S 20	Using MTX without taking precautionary action to prevent patient overdosing	9	8.4	++
S 1	Not using gastro-protection in oral NSAIDs/antiplatelets users at high risk of bleeding	8	8.2	++
S 3	Using oral NSAIDs in patients at increased risk of renal failure	8	7.9	++
S 21	Inconsistent monitoring of FBC in patients on DMARDs	8	7.8	++
S 27	Using sedatives, antipsychotics, anticholinergics in elderly patients	7	7.3	+
S 19	Using bone protection in users of long term oral corticosteroids	7	7.3	+
S 23	Excess duration of female hormones in patients at risk of gynaecological cancer	7	7.3	+
S 10	Excess duration of potassium supplements *and *potassium sparing diuretics	7	7.2	+
S 28	Using drugs to avoid in patients with heart failure	7	7.1	+
S 18	Excessive dosing of digoxin in patients susceptible to digoxin toxicity	7	7.1	+
S 24	Inconsistent monitoring of thyroid function in patients prescribed amiodarone	7	7.0	+
S 6	Inconsistent monitoring of U&Es in patients at risk of electrolyte imbalance	7	7.0	+
S 14	Co-prescribing beta blockers and rate-limiting calcium channel blockers	7	6.9	+
S 25	Using theophylline in elderly COPD patients without a compelling indication	7	6.9	+
S 15	Using beta blockers in patients with active asthma	7	6.8	+
S 13	Not using of laxatives in strong opioid users	7	6.8	+
S 29	Using drugs to avoid in children and young adults	7	6.7	+
S 5	Using COX II inhibitors in patients at high risk of cardiovascular events	7	6.6	+
S 7	Using thiazide diuretics in patients with a history of CKD	7	6.6	+
S 17	Using long acting sulphonylureas in patients at risk of hypoglycaemia	7	6.6	+
S 4	Using COX II inhibitors without compelling indication (low dose aspirin users)	7	6.4	+
S 16	Using metformin in patients with CKD	7	6.4	+
S 26	Excessive dosing of statins in patients on interacting drugs	7	6.3	+

	**Not scored as priorities for medication improvement**			

	***Quality***			
Q 13	Inadequate dose titration of ACEI, ARBs and BBs in chronic heart failure	6	6.2	
Q 1	Not using first line antihypertensives when initiating treatment for high blood pressure	6	6.4	
Q 18	Not using calcium/vitamin D supplementation in female elderly patients	6	6.4	
	***Safety***			
S 2	Using oral NSAIDs in the elderly without compelling indication (no previous trial of full dose paracetamol)	6	6.6	
S 9	Using of aldosterone antagonists in patients with a history of CKD	6	6.5	
S 11	Co-prescribing anti-infectives with high risk of affecting INR in patients on warfarin	6	6.4	
S 12	Using warfarin without a compelling indication in AF with low risk of stroke	6	6.3	
S 22	Using HRT in female patients at high risk of cardiovascular events	6	6.2	
S 8	Not using allopurinol in thiazide users with a history of gout	6	5.8	

Topics are ranked by median scores. Clusters of topics with the same median are ranked in descending order of mean score. Topics with a median of 8 or higher ('high priority') are coded '++' and those with a median of 7 ('priority') '+'.

## Discussion

This paper reports the development of a set of 176 explicit assessment criteria to identify patients at risk of PDRM from electronic data sources routinely held in UK primary care. The criteria set targets suboptimal selection, intensity or omissions of beneficial drug treatments (medication use quality) and high-risk use, inconsistent monitoring or patient instructions for drugs implicated in preventable harm (medication use safety) in primary care. All items are classified by clinical importance (appropriateness and necessity) as the output of an extended RAM process. Key professionals in UK primary care identified eleven clusters of thematically related medication assessment criteria (topics) as 'high priority' for quality improvement initiatives. The three highest rated topics related to methotrexate dosing instructions, high-risk prescribing of NSAIDs and antiplatelets and underuse of corticosteroids in asthma.

### Development process of the DQIP criteria set

The RAM approach had advantages over the Delphi technique as an initial step in the criteria development process, because the face-to-face meeting ensured the necessary commitment of panellists to place ratings on an extensive and thematically broad list of candidate criteria that were grounded in the evidence base. The original RAM approach was extended in this study by introducing the concept of 'necessary to avoid', in order to distinguish between inappropriate ('not worthwhile') and 'improper' medication use in safety terms (see table 1). As for the distinction between 'appropriate' and 'necessary', panellists required examples to apply and reason the concepts, but the absence of paradoxical 'appropriateness' and 'necessity' ratings is consistent with a reliable rating process.

A limitation of consensus methods such the RAM is that ratings may depend on panel composition [42]. The chosen panel combined clinical, public health and academic expertise in primary care medication use in general, rather than specialist expertise in the management of each medical condition covered. It is possible that generalists underestimate the implications of suboptimal medication use because they do not individually see relatively rare PDRM events that have significant impact at population level. Conversely, specialists tend to overestimate the importance of practices that fall within their own specialty [47,48]. However, since relatively few candidate criteria (22%) were rejected, it seems unlikely that including specialists would have substantially altered the results.

### Scope and focus of the DQIP criteria set

Consistent with the intended use of the DQIP criteria set, our literature search targeted commonly encountered medical conditions and drug groups implicated in PDRM events in primary care rather than exclusively focussing on the elderly. As a consequence, only 27% of all developed criteria are restricted to patients over 65 years with the majority of generated assessment criteria covering aspects of medication use which are not or not exclusively relevant to the elderly [8-10,49], such as primary prevention of vascular events, use of anti-diabetics in renal impairment [36] and treatments that are potentially harmful in children [36]. The fact that all topics identified as 'high priority' by the Delphi panel are age independent additionally underlines the relevance of not restricting a criteria set to be used in primary care to the elderly as is the case with many existing criteria sets [8-10,49].

A limitation of the medication assessment criteria developed for this study is that several established and potentially important criteria were not considered because the study focused on those that could be applied routinely to existing UK electronic clinical data. For example, international normalised ratio (INR) results in the UK are often held in bespoke systems which hinder the implementation of meaningful measures for monitoring anticoagulant use [1-4]. Similarly, although a broad spectrum of medication use categories are covered, the criteria set is mainly focussed on the prescribing and monitoring stages of the medication use process with minimal coverage of patient education and compliance. In the future, the increasing sophistication of clinical information systems and the ability to link clinical datasets with laboratory systems and dispensing data would make an even broader set of assessment criteria feasible.

Although the DQIP criteria set has been developed for application in UK primary care, the drug groups reported to be implicated in PDRM events in primary care are similar internationally [50-52], and we would expect the areas focused on to be relevant in other countries and health care settings. Nevertheless, some local adaptation may be required in order to account for differences in drug licensing, available resources, and clinical guidelines.

### Implications for quality improvement initiatives

The Delphi approach allowed stakeholders in primary care to prioritise the chosen medication use topics for improvement initiatives in UK primary care. The Delphi panel was deliberately chosen to include both day to day prescribers (GPs do almost all primary care prescribing, especially of the more complex kind being assessed in this study, but pharmacists prescribe for some patients and conditions) and those involved in prescribing governance and improvement (predominately pharmacists but including GPs with a more strategic role). A limitation is that our focus on professionals involved in primary care prescribing meant that we did not seek to include either specialist or patient/public perspectives in the Delphi panel. Since there is evidence that practitioners' perceptions of a targeted behaviour as meaningful is a pre-requisite to changing behaviour [53] we aimed to identify medication improvement topics which met this condition to inform the design of an intervention targeting primary care professionals.

It is important to note that even those topics that were not considered to be priorities (3 quality - and 6 safety topics) contain individual criteria that were agreed to be 'necessary' to do or avoid by the RAM panel. Examples are 'inadequate dose titration of ACEI, ARBs and beta blockers in chronic heart failure', and the 'using of warfarin without a compelling indication in atrial fibrillation with low risk of stroke'. These should therefore not be neglected. Lower priority ratings nevertheless indicate that changing and improving the corresponding medication use aspects may require targeted effort (or resources) in order to influence prescribing behaviour.

### Conclusions

The DQIP medication assessment criteria set presented here has been developed using established consensus methods and complements existing medication assessment instruments by not being limited to the elderly and by targeting a wide spectrum of medication use practices implicated in common and/or severe PDRM events in primary care. As all previously published explicit medication assessment tools, the criteria set presented here does not, however, provide comprehensive coverage of all situations that put patients at risk of PDRM, reflecting the large scope and high complexity of medication use in primary care and the limitations of current UK clinical information systems. The best choice of criteria set will therefore depend on the main purpose to be addressed and will be guided by local priorities. Informed by the priority ratings of a panel of UK primary care professionals, we have selected a subset of the DQIP criteria to serve as outcome measures in a cluster randomised trial evaluating the effectiveness of a complex intervention to improve prescribing safety (Trial registration number NCT01425502).

The DQIP criteria were primarily developed to facilitate the identification of patients at risk of PDRM from routine electronic data sets for a targeted review of their medication. However, we anticipate that they could also serve a range of other purposes, for example by informing the design of clinical decision support systems, where the classification of criteria by 'appropriateness' and 'necessity' may guide the selection of alerts that should or should not be interruptive to clinicians' workflow. Performance feedback is a further potential application, but in order not to overwhelm practitioners, the developed criteria are likely to require further prioritisation and/or the design of meaningful composites, for example by aggregating items that address the same topic [54] or medication use category [13].

An inherent limitation of explicit assessment criteria is that they cannot fully account for clinical factors that may justify deviations from what is considered to be best practice in an 'average' patient. The extent to which patients identified to be at risk of PDRM are judged by practitioners to represent actual opportunities for improvement (concurrent validity) and the extent to which any improvements in prescribing or monitoring translate into improved patient outcomes (predictive validity) therefore deserve further study.

## List of abbreviations

**ACEI: **Angiotensin converting enzyme inhibitor; **ACOVE: **Assessing care of vulnerable elders; **ACS: **Acute coronary syndrome; **ADE: **Adverse drug event; **AF: **Atrial fibrillation; **ARB: **Angiotensin receptor blocker; **BB: **Beta blocker; **BP: **Blood pressure; **CCB: **Calcium channel blocker; **CHADS_2_: **Score for stroke risk assessment in atrial fibrillation based on the following risk factors: cardiac failure, hypertension, age > 75, diabetes and stroke; **CHD: **Coronary heart disease; **CHD: **Coronary Heart Disease; **CHF: **Chronic heart failure; **CKD: **Chronic kidney disease; **CKD: **Chronic kidney disease; **COPD: **Chronic obstructive pulmonary disease; **COX: **Cyclo-oxygenase; **CVD: **Cardiovascular disease; **DM: **Diabetes mellitus; **DMARD: **Disease modifying antirheumatic drug; **DQIP: **Data driven quality improvement in primary care; **eGFR: **Estimated glomerular filtration rate; **FBC: **Full blood count; **GIP: **Gastro-intestinal protective agents; **GP: **General practitioner; **HbA1c: **Glycated haemoglobin; **HIV: **Human immunodeficiency virus; **HRT: **Hormone replacement therapy; **HTN: **Hypertension; **INR: **International normalised ratio; **IT: **Information technology; **MI: **Myocardial infarction; **MTX: **Methotrexate; **NSAID: **Non-steroidal anti-inflammatory drug (includes non-selective and COXII selective agents unless stated otherwise); **NS NSAID: **Non-selective NSAID **NYHA: **New York Heart Association; **PDRM: **Preventable drug related morbidity; **RAM: **RAND appropriateness method; **SABA: **Short acting beta 2 receptor agonist; **SD: **Standard deviation; **START: **Screening tool to alert doctors to right treatment; **STOPP: **Screening tool of older person's prescriptions; **TCA: **Tricyclic antidepressant; **TIA: **Transient ischaemic attack; **U&E: **Urea and electrolytes; **UCLA: **University College of Los Angeles; **UK: **United Kingdom; **Z-drug: **zopiclone, zolpidem or zaleplone.

## Competing interests

The authors declare that they have no competing interests.

## Authors' contributions

The study is part of the Data-driven Quality Improvement in Primary Care (DQIP) research programme, which is led by BG. TD led the literature review, conduct of RAM and Delphi studies, data analysis and wrote the first draft of the manuscript. All co-authors contributed to subsequent drafts. All authors have read and approved the final manuscript.

## Pre-publication history

The pre-publication history for this paper can be accessed here:

http://www.biomedcentral.com/1472-6904/12/5/prepub
